# Mesoporous silica nanoparticles as a drug delivery mechanism

**DOI:** 10.1515/biol-2022-0867

**Published:** 2024-05-15

**Authors:** Wei Zhang, Hongwei Liu, Xilong Qiu, Fanjiao Zuo, Boyao Wang

**Affiliations:** Tianjin Medical University Cancer Institute & Hospital, National Clinical Research Center for Cancer, Tianjin’s Clinical Research Center for Cancer, Key Laboratory of Cancer Prevention and Therapy, No. 1 West Huan-Hu Road, Ti Yuan Bei, Hexi District, Tianjin 300060, China; Department of Pharmacy, First Teaching Hospital of Tianjin University of Traditional Chinese Medicine, Tianjin, 300072, China; School of Chinese Materia Medica, Tianjin University of Traditional Chinese Medicine, No. 10 of Poyang Lake Road, Tuanpo Xincheng West District, Tianjin 301617, China; School of Traditional Chinese Medicine, Tianjin University of Traditional Chinese Medicine, Tianjin 301617, China

**Keywords:** drug slow-release system, controlled drug release system, mesoporous silica nanoparticles, review

## Abstract

Research in intelligent drug delivery systems within the field of biomedicine promises to enhance drug efficacy at disease sites and reduce associated side effects. Mesoporous silica nanoparticles (MSNs), characterized by their large specific surface area, appropriate pore size, and excellent biocompatibility, have garnered significant attention as one of the most effective carriers for drug delivery. The hydroxyl groups on their surface are active functional groups, facilitating easy functionalization. The installation of controllable molecular machines on the surface of mesoporous silica to construct nanovalves represents a crucial advancement in developing intelligent drug delivery systems (DDSs) and addressing the issue of premature drug release. In this review, we compile several notable and illustrative examples of MSNs and discuss their varied applications in DDSs. These applications span regulated and progressive drug release mechanisms. MSNs hold the potential to enhance drug solubility, improve drug stability, and mitigate drug toxicity, attributable to their ease of functionalization. Furthermore, intelligent hybrid nanomaterials are being developed, featuring programmable properties that react to a broad spectrum of stimuli, including light, pH, enzymes, and redox triggers, through the use of molecular and supramolecular switches.

## 1 Introduction

Drug delivery systems (DDSs) represent significant advancements in contemporary pharmaceutical research, reflecting improvements in dosage forms and formulation development. These systems stand at the vanguard of current scientific and technological progress, characterized by substantial advancements in theoretical foundations, innovative formulation designs, manufacturing methodologies, and clinical applications [1–3]. DDSs have the potential to enhance medication bioavailability by increasing solubility and regulating the rates of degradation and absorption [4,5]. Furthermore, by modifying medication molecules with biocompatible compounds to prevent recognition by the body’s immune system, these systems offer the potential to minimize drug-related adverse effects during medical treatment [6]. Nanomaterials have recently shown immense potential for use in biomedical fields, owing to the rapid advancement of nanotechnology and their exceptional physicochemical properties. Nanomaterials are extensively employed across various fields, including drug delivery [7], disease diagnostics and therapy [8,9], tissue engineering repair [10,11], among others, and have earned considerable recognition in the materials science domain for their contributions to controlled drug release systems. This acclaim is due to their inherent properties, which include easy synthesis, tunable size, chemical durability, large specific surface area, uniform and adjustable pore sizes, biocompatibility, surface modifiability, and effective loading capacity for poorly soluble pharmaceutical agents [12–14]. The rapid release of medications containing mesoporous silica nanoparticles (MSNs) is primarily attributed to the open pores of the MSNs.

The review provides an overview of MSNs, highlighting their advantages, slow-release, and controlled drug release system along with their applications. Our goal is to summarize recent research progress in mesoporous silica and then offer insights into strategies for achieving desired chemical properties using this nanoplatform for future practical applications. We aim to stimulate new ideas and inspire continued efforts in this emerging research field [15–17].

## 2 Mesoporous silica nanomaterials

Mesoporous materials, characterized by a porous structure with pore sizes ranging from 2 to 50 nm, have seen significant advancements in recent years. This progress is particularly notable in the development of two-dimensional hexagonal mesoporous silica, various transition metal oxides not based on silicon, and diverse nanoparticle assemblies. The outcome of related research efforts is the discovery of innovative materials with promising applications [18,19].

A significant breakthrough occurred in 1992 when scientists at the American Mobile Company developed the MCM-41 series of novel mesoporous silicon materials. They utilized a cationic alkyl quaternary ammonium salt surfactant as a template, employing the concept of liquid crystal templates [20]. MCM-41 mesoporous silica, one of these materials, has garnered considerable interest within the biomedical research community. Its appeal lies in its robust structural integrity, extensive specific surface area, pores organized in a uniform hexagonal pattern, ease of surface functionalization and modification, adjustable pore diameters, and low cytotoxicity [21,22].

## 3 Mesoporous silica sustained-release drug systems

**Figure 1:**
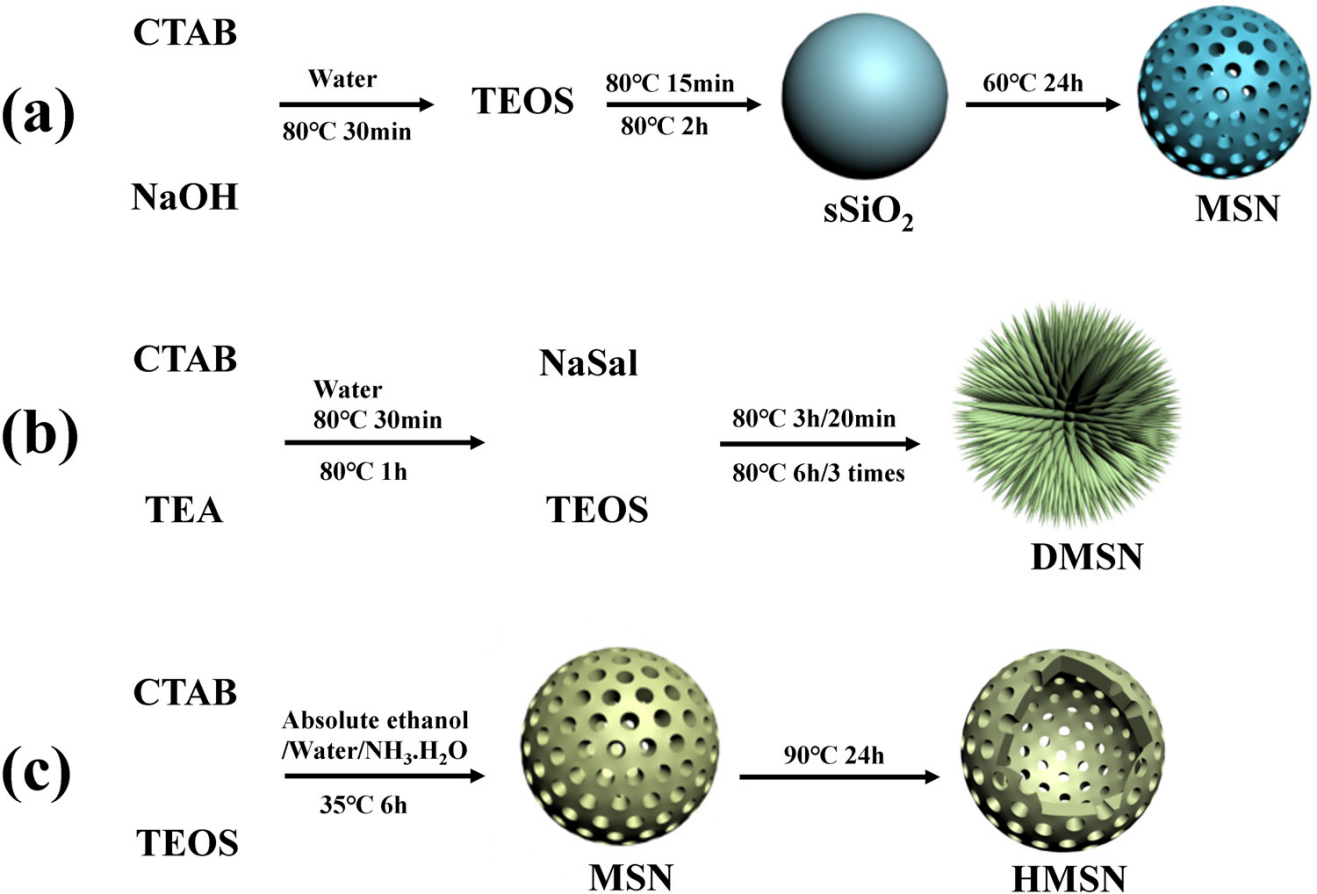
Schematic representation of the formation of MSN (a), DMSN (b), and HMSN (c), respectively.

Leveraging MSNs as a delivery method allows for the loading of hydrophobic drugs into MSN pores using non-aqueous solvents. As these drug molecules transition from the pores to the cytoplasm, the phospholipid bilayer facilitates their entry into the hydrophobic intracellular environment. Notably, drugs such as camptothecin [23] and paclitaxel [24], known to be encapsulated within MSNs, are effectively released from these pores. Drawing on the biocompatibility and cell permeability of MSNs, Wang et al. succeeded in encapsulating the drug telmisartan in mesoporous silica. The integration of these two elements during the formulation process enhanced both the bioavailability and dissolution rate of telmisartan [25]. Compared to raw drug materials, this approach markedly improved both the drug dissolution rates and cumulative drug dissolution. To boost the bioavailability of medications with low solubility and enhance their permeability, Wang et al. embarked on a study aimed at examining the effects of different drug loading methods on drug loading efficiency and dissolution rates within MSNs. Qiu et al. demonstrated the successful synthesis of MSNs, dendritic mesoporous silica nanoparticles (DMSNs), and hollow mesoporous silica nanoparticles (HMSNs), with all three types exhibiting higher drug loading and encapsulation efficiency. Analysis based on the first-order release equation curve and Higuchi equation parameters revealed that the loaded puerarin exhibited sustained-release properties [26], Figure 1.

## 4 Mesoporous silica controlled drug release system

MSNs possess a large specific surface area, excellent biocompatibility, and a stable skeletal structure, making them a viable option for drug delivery. This has spurred significant research interest. In 2004, Hernandez et al. ventured into the field of nanoscale-controlled valve mechanisms by introducing the groundbreaking concept of a nanovalve based on macrocyclic receptor molecules [27]. For the nanovalve to function as intended, three key components are necessary: a nanocontainer for carrying drugs, an actuator capable of moving chains in response to specific stimuli, and a suitable control strategy to govern this chain movement. Designing a DDS that responds to particular environmental stimuli is crucial for achieving controlled medication release within the body, thereby minimizing drug leakage and adverse effects on healthy tissues. Examples of stimuli include light [27], pH fluctuations [28], enzyme activity [29], redox reactions [30], and combinations of these factors [31].

### 4.1 Light-responsive controlled drug release systems

Leveraging light to trigger reactions offers numerous benefits, such as the ability for remote activation, non-invasiveness, precise control, and extensive applicability in the biomedical sector. UV-visible and near-infrared light serve as the primary light sources in DDS [32].

In a noteworthy experiment, Nguyen et al. utilized mesoporous silica coated with the photosensitive substance anthracene formic acid [33]. This molecule-coated surface was encased by a ring of tetravalent ionic cyclophane – CBPQT^4+^. Upon exposure to light, the photosensitive molecule formed a covalent bond with the CBPQT^4+^ ring, facilitating a light-induced electron transfer that ultimately resulted in the dissociation of the ring from the molecular bearing. This process enabled the development of a drug release mechanism responsive to light, simplifying the release of drug molecules from the mesoporous silica pores.

In a similar approach, Sierocki et al. incorporated azobenzene into the pores of mesoporous silica [34]. In its stable state, azobenzene adopts a trans configuration, which can be isomerized upon exposure to UV light. This exposure triggers the isomerization of azobenzene into the cis configuration. Further exposure to light at 450 nm induces continuous trans-cis and cis-trans photoisomerization of azobenzene. The oscillatory movements of the detached azobenzene head, resulting from this series of changes, trigger the release of drug molecules into the surrounding solution from the mesoporous silica pores.

Additionally, Li et al. employed host–guest interactions to attach trans-cinnamamide to the molecular bearing linked to the mesoporous silica surface, utilizing cucurbit[7]uril (CB[7]) [35]. Upon exposure to UV radiation at 300 nm, the trans-cinnamamide underwent isomerization into the cis structure. This isomerization induced spatial repulsion caused CB[7] to detach from the molecular bearing, thereby opening the mesoporous silica pores and allowing the release of drug molecules.

### 4.2 pH-responsive controlled drug release systems

pH-sensitive drug delivery methods are gaining prominence due to their capacity to release drugs in response to the varying acidic or alkaline environments present in different parts of the body, which have distinct pH levels [36,37]. This characteristic enhances therapeutic efficacy while minimizing adverse impacts on non-target tissues. A pH-responsive nanovalve system is designed to selectively release drugs at tumor sites, leveraging the pH differential between tumor tissues (typically pH 5.8–7.1) and normal tissues (pH 7.4) within the body’s microenvironment [38]. This targeted approach facilitates cancer treatment without negatively affecting healthy tissues.

Stoddart et al. explored the interaction of CB[6] with amino groups on the molecular bearings of mesoporous silica as a potential drug release method. This interaction allows for binding or detachment based on pH variations. Under neutral and acidic conditions, the CB[6] ring securely encases the molecular bearings adorned with bisquaternary ammonium groups on the mesoporous silica surface, effectively trapping drug molecules within the mesoporous silica pores. Under alkaline conditions, deprotonation of the molecular bearings occurs, breaking the CB[6] binding and thereby enabling the release of drug molecules from the mesoporous silica [38].

Yang et al. investigated a pH-responsive, mesoporous silica-based drug release system functionalized with column[5] aryl hydrocarbon (CP[5]A) [39]. In this system, rotaxane molecules on the mesoporous silica surface are encased by electron-rich CP[5]A, effectively retaining drug molecules within the pores of the mesoporous silica under alkaline or neutral conditions. However, in acidic environments, CP[5]A dissociates from the rotaxane molecules, facilitating the easier release of drug molecules from the mesoporous silica.

**Figure 2:**
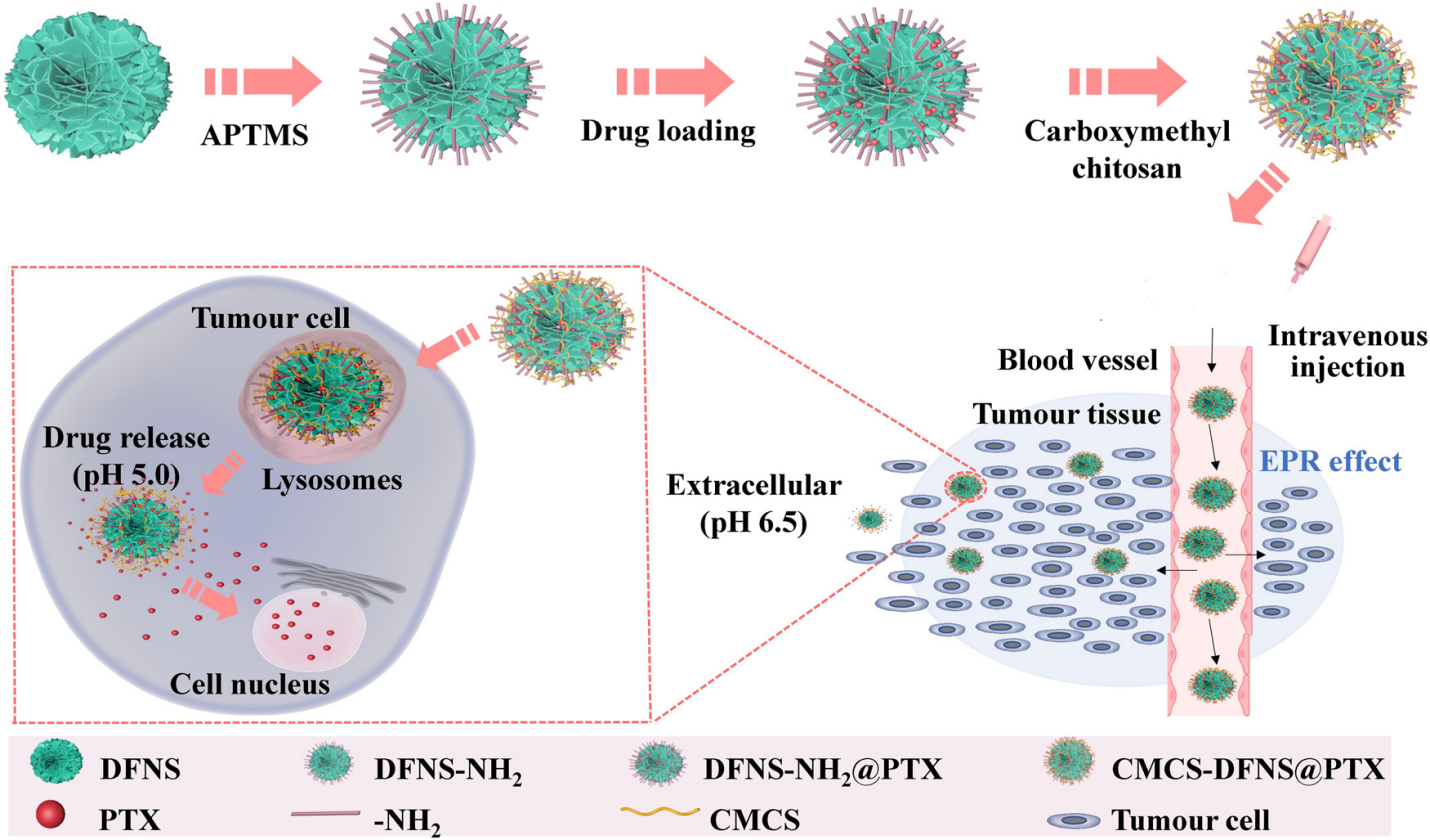
Schematic illustration of the synthesis process of CMCS-DFNS@PTX and the pH-responsive release.

Oiu et al. developed a method for delivering paclitaxel using carboxymethyl chitosan (CMCS)-functionalized dendritic fibrous nano-silica (DFNS). DFNS, characterized by a three-dimensional dendritic structure, was synthesized using a double-template method. A pH-responsive, nanoloaded vehicle system was then prepared by grafting CMCS onto DFNS through a covalent binding reaction. *In vitro* cellular experiments indicated that CMCS-DFNS significantly enhanced drug uptake efficiency in breast cancer MCF-7 cells. Crucially, *in vivo* and cellular pharmacokinetic results demonstrated that CMCS-DFNS could prolong circulation time and increase the relative bioavailability of paclitaxel. Therefore, this pH-responsive DDS holds potential for the delivery of anti-tumor drugs and offers a new pathway for enhancing the bioavailability of other compounds with low inherent bioavailability [40], Figure 2.

### 4.3 Enzyme-responsive controlled drug release systems

The variability in enzymes across different tissues in the human body highlights the importance of employing biomolecules as gatekeepers for controlled drug release within mesoporous systems *in silico*. E.g., a range of biomolecules – including antigen–antibody interactions [41], hybridized single-stranded DNA [42], enzymes [43], sugars [44], ribose [45–48], and proteins [49] have been engineered to function as gating elements at the entrances of mesoporous silica structures. This approach confers a high level of specificity, precision, and efficiency on the resultant mesoporous silica drug release systems.

In 2008, Stoddart et al. introduced a mesoporous silica drug release system designed to respond to enzymatic stimulation [50]. In this system, α-cyclodextrin (α-CD) wraps around the polyethylene glycol molecular bearings on the surface of the mesoporous silica, forming a bond with the upper ester linkage and thus securing drug molecules within the mesoporous silica pores. Upon introduction of porcine heparan esterase, which catalyzes the enzymatic hydrolysis of the ester bond, the molecular bearings break apart. This causes α-CD to detach from them, thereby facilitating the release of the drug from the pores.

Yang et al. developed organic–inorganic hybrid nanomaterials that leverage supramolecular interactions between hosts and guests. These materials not only showcase excellent biocompatibility but also exceptional controlled release capabilities in response to specific external stimuli. The release mechanisms, precisely regulated by cup-shaped aromatic-based nanogate systems enable the responsive release of acetylcholine – a neurotransmitter – into the environment. This approach facilitates timed, targeted, and quantifiable controlled release, exemplifying a significant advancement in the field of DDSs [51].

### 4.4 Redox-responsive controlled drug release systems

Recently, notable progress has been made in the development of controlled drug release systems that react to alterations in the body’s redox environment. Studies have shown that the unique reducing conditions present within tumors act as a distinct trigger for the decomposition of redox-responsive delivery systems inside tumor cells, enabling the release of encapsulated medication. These redox-responsive systems present several benefits, such as stability in normal tissues and enhanced therapeutic efficacy compared to other controlled release mechanisms. Consequently, this can significantly diminish the toxicity and adverse effects associated with both the drugs and the nanocarriers [30,52].

Zink et al. developed molecular bearings known as rotaxanes, which were attached to the surface of mesoporous silicon. These rotaxanes featured a bistable state with two attachment sites: tetrathiafulvalene (TTF) and dioxynaphthalene (DNP). A CBPQT^4+^ ring, serving as a valve molecule, was positioned around the molecular bearings in their bistable state, demonstrating a strong affinity for TTF. In the absence of an oxidizing reagent, CBPQT^4+^ remained attached to the TTF site, distanced from the mesoporous silica pore, thus allowing the ingress or egress of drug molecules. Upon the introduction of Fe(ClO_4_)_3_·6H_2_O, TTF was oxidized to TTF^2+^, prompting the CBPQT^4+^ to migrate to the DNP site close to the pore entrance, effectively encapsulating the drug molecules within. The subsequent reduction of TTF to its original state by ascorbic acid caused CBPQT^4+^ to revert to the DNP site, facilitating controlled drug release [53].

Zink et al. further innovated by modifying the surface of mesoporous silicon with ferrocene dicarboxylic acid and β-cyclodextrin (β-CD) to engineer nanovalves. Upon application of an oxidizing voltage via cyclic voltammetry, the resulting positively charged oxidized ferrocenyl salt disrupted the stable inclusion complex formation with β-CD. This process led to the oxidation of the ferrocenyl dicarboxylic acid, prompting β-CD to detach, which in turn facilitated the release of drug molecules contained within the pore channels.

Zhao et al. utilized electrostatic interactions to attach negatively charged single-stranded DNA to positively charged disulfide bonds on the surface of mesoporous silicon. The introduction of reducing agents such as dithiothreitol and glutathione resulted in the cleavage of the disulfide bonds, enabling the single-stranded DNA to detach and thereby release the drug. Importantly, the intracellular concentration of the reducing agent glutathione is significantly higher (up to 10 mM) compared to extracellular concentrations (less than 10 µM). This difference in glutathione levels makes mesoporous silica an ideal storage microenvironment outside cells, offering considerable promise for the targeted delivery of therapeutic drugs, particularly in cancer treatment [54].

## 5 Challenges and opportunities

Leveraging the distinctive attributes of MSNs, multifunctional MSN nanocomposites emerge as potent agents for diverse applications. Nonetheless, significant opportunities remain to enhance the commercial production of MSNs. To boost yield, the development of new materials and synthetic strategies is crucial, particularly with a focus on efficiently synthesizing ultra-large-sized MSNs. Moreover, concerted efforts are required to ingeniously design smart theranostics that facilitate highly effective therapy under remote control and to establish correlations between experimental outcomes and therapeutic efficacy. This approach will pave the way for real-time monitoring of therapeutic results, thereby advancing personalized theranostics. The ongoing development of MSN-based theranostics for biomedical applications remains a vibrant and critical area of research. Continued in-depth investigation into MSNs promises to yield exciting and promising advancements for the chemistry and biomedicine communities by contributing novel discoveries and addressing existing challenges. Interdisciplinary collaboration is paramount to further refine the performance of MSN-based theranostics and ensure their successful clinical implementation.

## 6 Future trends

In conclusion, our review has highlighted recent advancements in MSNs that have led to the creation of novel smart materials with extensive applications across various fields. MSNs have been endowed with numerous advantageous properties, establishing them as superior solid supports for constructing intelligent machinery. Significant strides have been made in developing biocompatible nanocarriers capable of controlled entrapment and release of cargo molecules in response to a wide array of internal and external stimuli. Consequently, MSNs hold a promising future in the pharmaceutical industry, presenting a broad spectrum of potential applications.
